# TIPIC syndrome in a patient following sorafenib treatment for acute myeloid leukemia: a rare case report

**DOI:** 10.3389/fonc.2024.1484256

**Published:** 2024-10-31

**Authors:** Chang Chen, Jinman Zhong, Wanzhen Hu, Jiewen Tan, Dan Xiong

**Affiliations:** Departments of Hematology, Shunde Hospital, Southern Medical University (The First People’s Hospital of Shunde), Foshan, Guangdong, China

**Keywords:** transient perivascular inflammation of the carotid artery syndrome (TIPIC), acute myeloid leukemia, FLT3, sorafenib, VEGFR

## Abstract

Transient Perivascular Inflammation of the\ Carotid Artery (TIPIC) syndrome is uncommon, and cases of TIPIC induced by the targeted drug, sorafenib, are extremely rare. This case report describes a patient with acute myeloid leukemia carrying an FMS‐like tyrosine kinase 3 mutation, who developed TIPIC syndrome, which may have been induced by sorafenib treatment. A 65-year-old woman diagnosed with acute myeloid leukemia experienced severe neck pain and sclerotic blisters on her palms and soles during sorafenib treatment. Carotid ultrasound revealed thickening of the right common carotid artery (RCCA) wall, and magnetic resonance imaging revealed perivascular tissue edema in the distal RCCA. Following clinical and imaging assessments, the patient was diagnosed with TIPIC syndrome. Treatment involved a one-week course of oral steroid therapy with dexamethasone and non-steroidal anti-inflammatory drugs, which led to complete clinical recovery. TIPIC syndrome involves transient nonspecific perivascular inflammation of the carotid adventitia; however, the precise underlying cause remains unclear. In this study, we report a rare case and explore the potential pathophysiological mechanisms through a review of the existing literature.

## Introduction

Transient Perivascular Inflammation of the Carotid Artery (TIPIC) is a rare disease with an extremely low incidence. It is characterized by transient nonspecific perivascular inflammation of the carotid adventitia. Owing to the uncommon nature of TIPIC, its etiology is not well known. To the best of our knowledge, TIPIC syndrome has not been reported in sorafenib-treated patients with acute myeloid leukemia. To address this gap, we report this case and conduct a literature review to deepen our understanding of this rare disease and investigate the potential mechanisms underlying sorafenib-induced TIPIC syndrome. Our aim was to provide valuable insights for clinical practice.

## Case presentation

**Figure 1:**
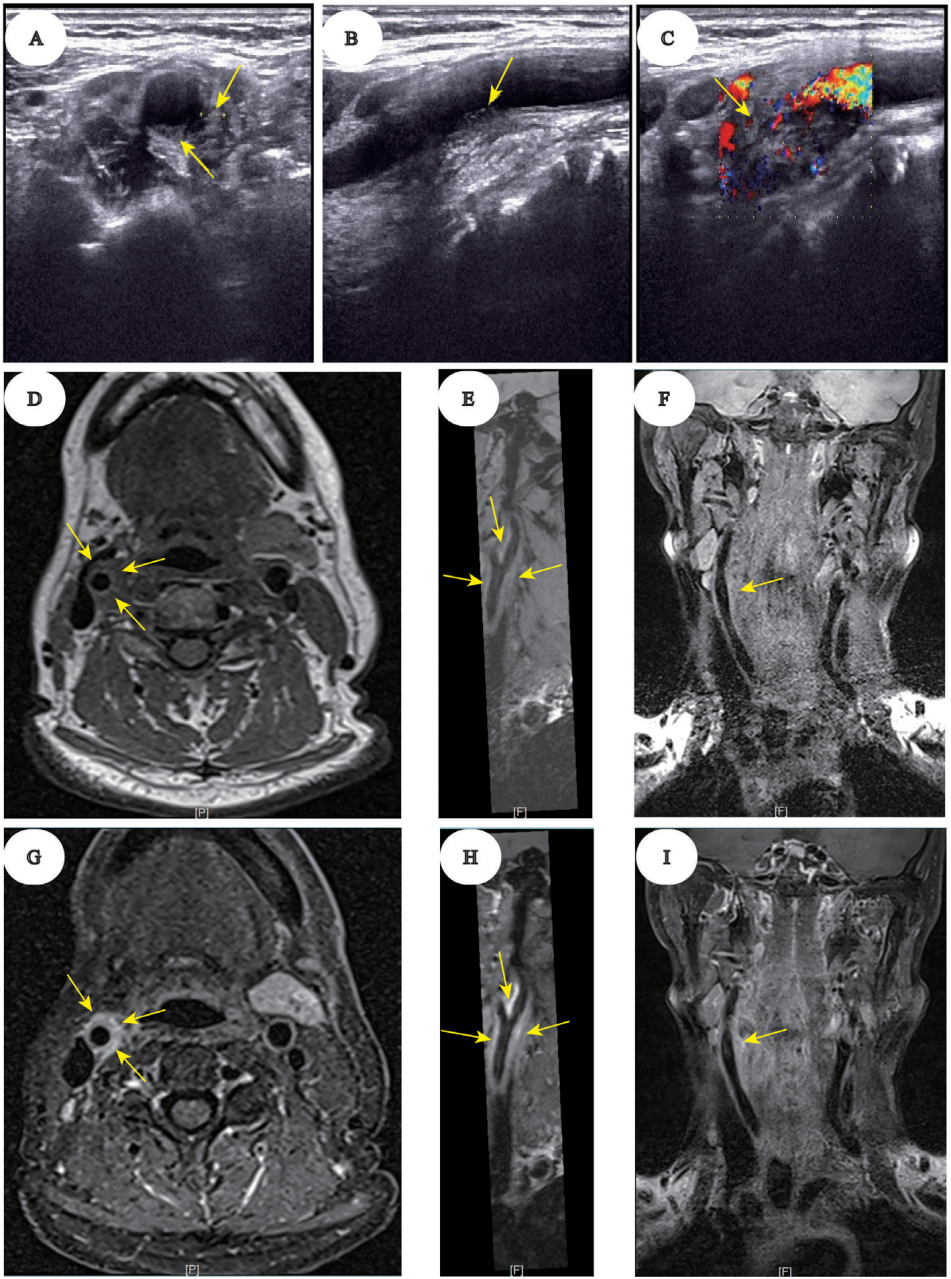
Ultrasound and MRI imaging of right carotid artery wall thickening in TIPIC syndrome. Ultrasound imaging in axial **(A)**, sagittal **(B)**, and coronal **(C)** planes. Eccentric thickening of the arterial wall is observed from the upper right CCA to the bifurcation, particularly on the posteromedial wall. The boundary between the arterial wall and surrounding tissue is indistinct. Pre-contrast MRI vessel wall imaging in axial **(D)**, sagittal **(E)**, and coronal **(F)** planes. Mild luminal narrowing and uniform wall thickening are seen in the mid-to-distal segments of the right CCA, extending into the ICA. Post-contrast MRI vessel wall imaging in axial **(G)**, sagittal **(H)**, and coronal **(I)** planes. Significant homogeneous enhancement of the thickened vessel walls is evident.

A 65-year-old woman was diagnosed with acute myeloid leukemia (AML-M2a subtype) on July 18, 2023, accompanied by mutations in FMS‐like tyrosine kinase 3 (*FLT3*)-ITD, NPM1, TET2, EZH2, and other genes. However, the patient had a medical history of hypothyroidism and hypertension, which were managed with thyroxine replacement and antihypertensive therapy. Moreover, the patient consented to undergo a single cycle of decitabine, aclarubicin, cytarabine, and granulocyte colony-stimulating factor (D-CAG) regimen as induction chemotherapy. In addition, subsequent evaluation revealed complete remission (CR) morphologically, with negative minimal residual disease (MRD) testing by flow cytometry. Following this, the patient underwent three additional cycles of the same D-CAG regimen as consolidation chemotherapy. However, the patients’ *FLT3* quantitative analysis consistently indicated positivity, with fluctuations between 23.7% and 30.3%. To achieve a deeper molecular response, sorafenib, an *FLT3* inhibitor, was initiated as a targeted therapy on December 13, 2023. Additionally, D-CAG regimen was used on December 13, 2023 and the last time granulocyte colony-stimulating factor (G-CSF) was used in this cycle of chemotherapy was on December 28, 2023. The administration of sorafenib was temporarily halted during the period of myelosuppression following chemotherapy, attributed to neutropenia. Upon discharge, the patient experienced a recovery in bone marrow hematopoiesis and subsequently resumed sorafenib treatment on January 6, 2024. After initiating sorafenib treatment, the patient was readmitted to the hospital on January 10, 2024, presenting with acute, persistent, and severe pain localized to the right side of the neck. This was accompanied by symptoms consistent with hand-foot syndrome, characterized by sclerosing blisters on both the palms and soles. The schematic diagram for the patient timeline information is shown (**Supplementary Figure 1**). Physical examination revealed tenderness in the right cervical region and the absence of palpable swelling or local skin infection and damage. A series of neurologic assessments were normal, including mental status, muscle strength, sensory, reflexes, and autonomic nervous system. The patient had no recent history of cervical trauma, vascular disease, or infection. Complete blood count, inflammatory marker such as C-reactive protein and autoimmune vasculitis antibodies were found to be normal. Cervical vascular Doppler ultrasound is widely used in clinical examinations owing to its convenience. Meanwhile, high-resolution vessel wall MRI has proven to be an optimal method of directly displaying the features of vascular diseases. Given these advantages, we opted for cervical vascular Doppler ultrasound (Equipment brand: HITACHI, type: ARIETTA 70) and magnetic resonance angiography (Equipment brand: SIEMENS, type: KYRA 3.0 T) to investigate the etiology of neck pain (1). Doppler ultrasound scan of the neck revealed eccentric thickening of the arterial wall extending from the upper right common carotid artery (CCA) to the bifurcation, particularly on the posteromedial wall. The boundary between the arterial wall and surrounding tissue was indistinct. MRI images revealed mild luminal narrowing and uniform wall thickening in the mid-to-distal segments of the right CCA, extending into the internal carotid artery (ICA). Additionally, post-contrast MRI images showed significant homogeneous enhancement of the thickened vessel walls (**Figure 1**). Therefore, the patient was diagnosed with TIPIC syndrome based on clinical and imaging findings. We suspected TIPIC due to sorafenib, so we stopped the medication immediately. Treatment consisted of oral steroid therapy with dexamethasone (5 mg/day) and non-steroidal anti-inflammatory drugs (NSAIDs; celecoxib capsules, 200 mg, twice daily) for one-week, leading to complete clinical recovery.

## Discussion

TIPIC syndrome was first described by Fay in 1927 (2). It typically presents as an acute-onset, intense, and self-limiting pain overlying the carotid artery, often radiating to adjacent areas. Radiological findings indicated perivascular inflammation of the carotid sheath and adventitia. Ultrasound and MRI are crucial for demonstrating periarterial and intravascular changes; therefore, imaging is important for diagnosing TIPIC (2–7). A positive response to NSAIDs or a short course of corticosteroids helps confirm the diagnosis. The case presented in this report aligns with the diagnostic criteria for TIPIC, as evidenced by the clinical presentation and imaging findings. Therefore, our results suggested that sorafenib administration may precipitate this development. Upon review of the existing literature, it was determined that this particular trigger has not been previously documented.

Although the diagnosis of TIPIC is not particularly difficult, its underlying etiology and pathogenesis remain unclear. Thus, the current literature proposes some hypothesized etiologies for infectious and autoimmune diseases based on imaging manifestations and limited histological findings (7), with the most widely accepted theory being inflammatory diseases of the carotid artery wall. A case report by Peter (8) described a patient with TIPIC who underwent right carotid endarterectomy, and histopathological biopsy revealed vascular proliferation, fibroblast proliferation, and low-grade chronic active inflammation. Multiple etiological factors have been identified as being linked to TIPIC syndrome, including upper respiratory tract infections, high-altitude exposure, lymphadenopathy, traumatic vascular diseases, autoimmune diseases, thyroiditis, large vessel vasculitis, arterial dissections, and thromboses (9, 10). Additionally, reports have suggested that granulocyte colony-stimulating factor (11) and traditional chemotherapy drugs (12) may trigger TIPIC (13). In our case, The patient was diagnosed with acute leukemia and achieved CR. She had previously undergone four cycles of D-CAG chemotherapy without exhibiting any signs of TIPIC, and G-CSF administration was ceased over 10 days prior to the onset of symptoms. This temporal relationship suggests that traditional chemotherapy agents and G-CSF are improbable contributors to the development of TIPIC. Additionally, both C-reactive protein and immune vasculitis assays returned negative results, effectively excluding autoimmune vasculitis and infection as potential etiological factors. Moreover, the temporal correlation between the emergence of hand-foot skin reactions and neck pain subsequent to medication use suggests a potential causal relationship between sorafenib administration and the observed TIPIC.

Sorafenib is an orally administered multitarget tyrosine kinase inhibitor that exerts antitumor effects by inhibiting angiogenesis and tumor cell proliferation through targeting the vascular endothelial growth factor receptor (VEGFR) family. The vascular endothelial growth factor receptor 2 (VEGFR2) serves as the principal receptor mediating the biological effects of vascular endothelial growth factor (VEGF) and is essential for processes such as angiogenesis, vascular permeability, and endothelial cell survival (14–17). When sorafenib exerts its effects by inhibiting VEGFR2, it effectively blocks the signaling pathways initiated by the binding of VEGF to its receptor, thereby attenuating the proliferation, migration, and angiogenesis of endothelial cells. Given that VEGFR2 also contributes to the maintenance of normal vascular endothelial cells, the administration of sorafenib may result in direct damage to these cells. This damage includes the inhibition of cell proliferation, a reduction in cell migratory capacity, and the disruption of vascular integrity (18). Drugs such as bevacizumab and axitinib (19), which function as VEGFR2 inhibitors, exert their effects on endothelial cells by obstructing the interaction between vascular endothelial growth factor (VEGF) and its receptor VEGFR2. This inhibition results in a cascade of outcomes, including diminished angiogenesis, reduced vascular permeability, and the induction of endothelial cell apoptosis. Theoretically, they could all trigger TIPIC. In addition to its action on VEGFR2, sorafenib influences a range of receptors and signaling pathways associated with tumor growth, angiogenesis, and tumor cell survival. Notably, it also inhibits VEGFR1 and VEGFR3, both of which play significant roles in angiogenesis (20). As sorafenib can block various targets in the VEGF pathway (21), it may disrupt vascular integrity, leading to endothelial dysfunction (17). The endothelium regulates vascular tone and blood flow, and maintains vascular homeostasis (22). Therefore, we hypothesize that this complex network of mechanisms hinders the normal vascular repair process, thereby increasing susceptibility to vascular inflammation.

**Figure 2:**
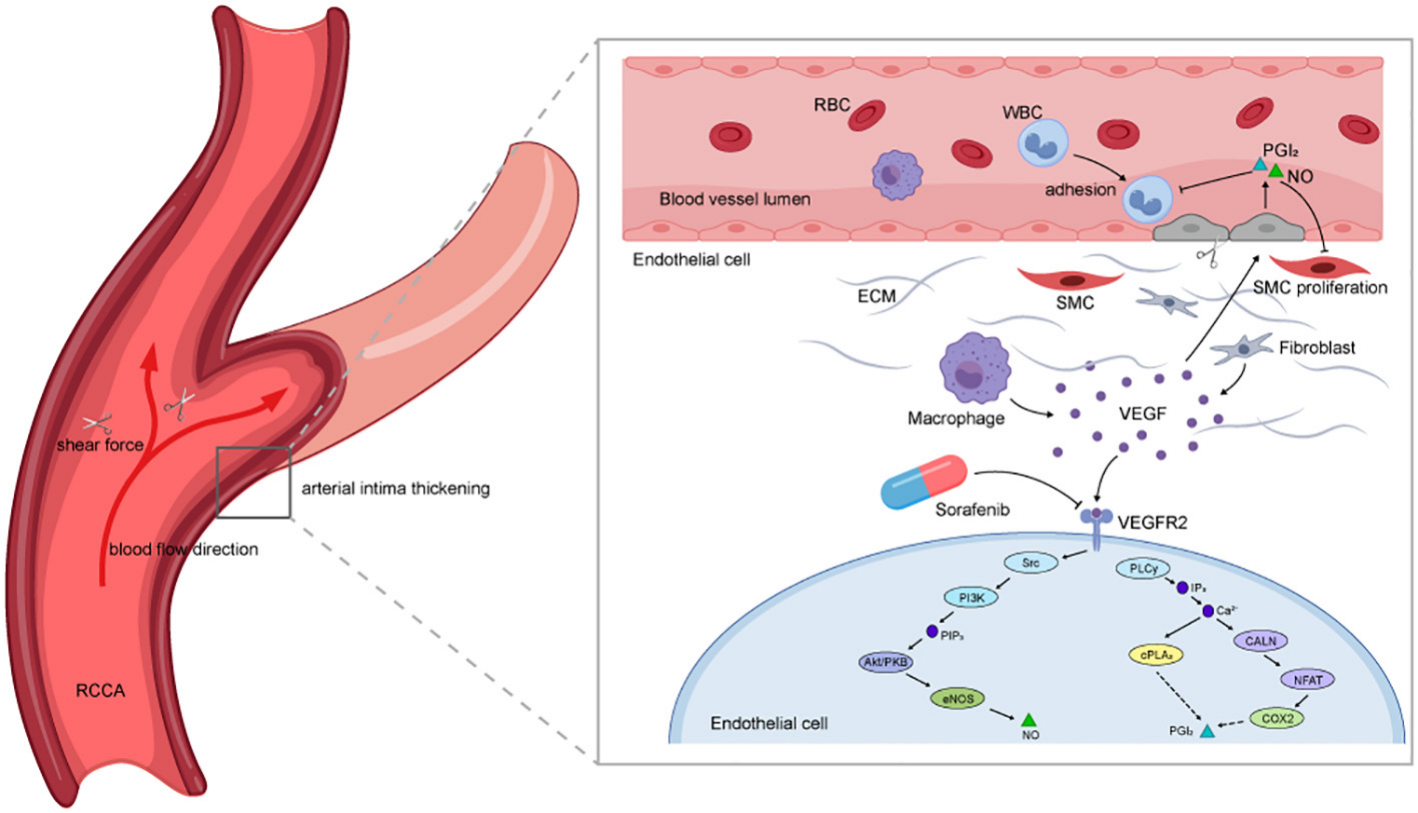
Diagram of the potential mechanisms by which sorafenib contributes to TIPIC. The diagram illustrates how sorafenib may induce TIPIC through its pharmacological effects and hemodynamic factors. The curved red arrows represent the direction of blood flow, while the scissors symbolize the shear stress acting on the vessel wall. Together, these elements highlight how local hemodynamic forces, combined with sorafenib’s inhibition of endothelial function, contribute to vascular inflammation.

Why does TIPIC predominantly occur in the carotid artery? High shear stress and its spatial gradient around arterial bifurcations are thought to induce endothelial dysfunction (23). Vascular endothelial cells (ECs) form a monolayer on the luminal walls of blood vessels and are continually exposed to hemodynamic stresses, such as shear stress (SS) generated by blood flow (23). LaMack and Friedman suggested that ECs can sense shear gradients (SSG) and SS independently (24). The magnitude of the relationship between SS and SSG plays an important role in regulating morphological changes in ECs. When their proportion is imbalanced, ECs exhibit inflammatory responses when they cannot adapt their morphology to flow (23, 25), resulting in vascular endothelial dysfunction. Our hypothesis suggests that, in combination with the anti-VEGFR pharmacological mechanism of sorafenib, these factors may collectively contribute to the development of TIPIC (**Figure 2**).

Interestingly, sorafenib-induced TIPIC syndrome appears to differ from the idiopathic form in both presentation and potential natural history. Sorafenib’s inhibition of multiple kinases involved in endothelial function, notably VEGFR2, can lead to widespread endothelial dysfunction and inflammation. Unlike idiopathic TIPIC, which typically involves the carotid bulb and proximal internal carotid artery (ICA) due to localized hemodynamic stress, sorafenib-induced endothelial injury may extend inflammation to the common carotid artery (CCA), as observed in our patient. This broader vascular involvement may be due to sorafenib’s systemic effects on endothelial cells, altering vascular homeostasis and increasing susceptibility to inflammation along the entire carotid artery. Furthermore, the patient’s comorbidities, such as hypertension and previous chemotherapy, might have compounded the endothelial injury, predisposing the CCA to inflammation. While the natural history of idiopathic TIPIC is generally self-limiting, the continuation of sorafenib could theoretically lead to a more protracted or recurrent course due to ongoing endothelial disruption. In our case, discontinuation of sorafenib resulted in symptom resolution within 14 days, suggesting that timely identification and management are crucial. These observations highlight the need for clinicians to consider drug-induced vascular inflammation in patients receiving multikinase inhibitors or other anti-angiogenic targeted therapies, especially when they present with atypical imaging findings. Awareness of this potential adverse effect can lead to prompt intervention, potentially preventing prolonged morbidity.

Herein, we present a case of sorafenib-induced TIPIC. Given the widespread use of sorafenib in targeted therapy for various tumors, it is imperative to increase awareness of TIPIC as a potential adverse reaction. Further research on the pathogenesis of TIPIC is warranted for a more comprehensive understanding of this condition.

## Data Availability

The original contributions presented in the study are included in the article/**Supplementary Material**, further inquiries can be directed to the corresponding author.

## Supplementary material

Supplementary Figure 1 The schematic diagram for the patient timeline information.

## References

[1] Sui B Gao P . High-resolution vessel wall magnetic resonance imaging of carotid and intracranial vessels. Acta Radiol. (2019) 60:1329–40. doi: 10.1177/0284185119826538 30727746

[2] Micieli E Voci D Mumoli N Mastroiacovo D Grigorean A Obadia M . Transient perivascular inflammation of the carotid artery (TIPIC) syndrome. Vasa. (2022) 51:71–7. doi: 10.1024/0301-1526/a000989 35130715

[3] Ulus S Aksoy Ozcan U Arslan A Buturak A Dincer A Kara S . Imaging spectrum of TIPIC syndrome: validation of a new entity with vessel wall imaging. Clin Neuroradiol. (2020) 30:145–57. doi: 10.1007/s00062-018-0746-5 30470848

[4] Venetis E Konopnicki D Jissendi Tchofo P . Multimodal imaging features of transient perivascular inflammation of the carotid artery (TIPIC) syndrome in a patient with Covid-19. Radiol Case Rep. (2022) 17:902–6. doi: 10.1016/j.radcr.2021.12.005 PMC8758192 35043074

[5] Rafailidis V Chryssogonidis I Tegos T Partovi S Charitanti-Kouridou A Staub D . Role of multi-parametric ultrasound in transient perivascular inflammation of the carotid artery syndrome. Ultrasound. (2019) 27:77–84. doi: 10.1177/1742271x18822658 31037091 PMC6475971

[6] Mathangasinghe Y Karunarathne RU Liyanage UA . Transient perivascular inflammation of the carotid artery; a rare cause of intense neck pain. BJR Case Rep. (2019) 5:20190014. doi: 10.1259/bjrcr.20190014 31938559 PMC6945257

[7] Peycheva M Zdravkova T Zlatareva D Viteva E Harizanova Z Meinel TR . Transient perivascular inflammation of the carotid artery-A transient but potentially recurrent disease. Clin Case Rep. (2024) 12:e8322. doi: 10.1002/ccr3.8322 38250093 PMC10797210

[8] Upton PD Smith JG Charnock DR . Histologic confirmation of carotidynia. Otolaryngol Head Neck Surg. (2003) 129:443–4. doi: 10.1016/s0194-59980300611-9 14574303

[9] Kamath N Kharkhele R Waghela R Aggrawal A Mathew J Padiyar S . Transient perivascular inflammation of the carotid artery (TIPIC) syndrome - a rare differential for anterior neck pain - Series of 3 cases and review of literature. Mod Rheumatol Case Rep. (2024) [Online ahead of print]. doi: 10.1093/mrcr/rxae031 38780237

[10] Castro A Zerpa F Gesner L . Tips for transient perivascular inflammation of the carotid artery syndrome. J Emerg Med. (2024) 67:e60–e4. doi: 10.1016/j.jemermed.2024.01.013 38825530

[11] Arnould B Miranda S Mignon F Camus V . G-CSF-induced TIPIC syndrome and large vessel vasculitis: A case report. Clin Case Rep. (2023) 11:e7918. doi: 10.1002/ccr3.7918 37720704 PMC10502199

[12] Hayashi S Maruoka S Takahashi N Hashimoto S . Carotidynia after anticancer chemotherapy. Singapore Med J. (2014) 55:e142–4. doi: 10.11622/smedj.2014127 PMC4293954 25273942

[13] Choudhary N Gupta V Mishra P Banerjee M . Radiological presentation of transient perivascular inflammation of carotid artery syndrome in a patient with myelodysplasia. Neuroradiol J. (2024) 37:126–7. doi: 10.1177/19714009231166079 PMC10863575 36951500

[14] Liu R Meng Y Zhu M Zhai H Lv W Wen T . Study on novel PtNP-sorafenib and its interaction with VEGFR2. J Biochem (2021) 170:411–7. doi: 10.1093/jb/mvab053 33944931

[15] Ai L Xu Z Yang B He Q Luo P . Sorafenib-associated hand-foot skin reaction: practical advice on diagnosis, mechanism, prevention, and management. Expert Rev Clin Pharmacol. (2019) 12:1121–7. doi: 10.1080/17512433.2019.1689122 31679411

[16] Abdelgalil AA Alkahtani HM Al-Jenoobi FI . Sorafenib. Profiles Drug Subst Excip Relat Methodol. (2019) 44:239–66. doi: 10.1016/bs.podrm.2018.11.003 31029219

[17] Li J Zhang L Ge T Liu J Wang C Yu Q . Understanding sorafenib-induced cardiovascular toxicity: mechanisms and treatment implications. Drug Des Devel Ther. (2024) 18:829–43. doi: 10.2147/dddt.S443107 PMC10959117 38524877

[18] Mabeta P Steenkamp V . The VEGF/VEGFR axis revisited: implications for cancer therapy. Int J Mol Sci. (2022) 23:15585. doi: 10.3390/ijms232415585 36555234 PMC9779738

[19] Hirsch L Flippot R Escudier B Albiges L . Immunomodulatory roles of VEGF pathway inhibitors in renal cell carcinoma. Drugs. (2020) 80:1169–81. doi: 10.1007/s40265-020-01327-7 32601914

[20] Zachary I Gliki G . Signaling transduction mechanisms mediating biological actions of the vascular endothelial growth factor family. Cardiovasc Res. (2001) 49:568–81. doi: 10.1016/s0008-6363(00)00268-6 11166270

[21] Weinberg RA . The molecular basis of oncogenes and tumor suppressor genes. Ann N Y Acad Sci. (1995) 758:331–8. doi: 10.1111/j.1749-6632.1995.tb24838.x 7625701

[22] Vanhoutte PM Shimokawa H Feletou M Tang EH . Endothelial dysfunction and vascular disease - a 30th anniversary update. Acta Physiol (Oxf). (2017) 219:22–96. doi: 10.1111/apha.12646 26706498

[23] Yoshino D Sakamoto N Sato M . Fluid shear stress combined with shear stress spatial gradients regulates vascular endothelial morphology. Integr Biol (Camb). (2017) 9:584–94. doi: 10.1039/c7ib00065k 28548171

[24] LaMack JA Friedman MH . Individual and combined effects of shear stress magnitude and spatial gradient on endothelial cell gene expression. Am J Physiol Heart Circ Physiol. (2007) 293:H2853–9. doi: 10.1152/ajpheart.00244.2007 17766484

[25] Dai G Kaazempur-Mofrad MR Natarajan S Zhang Y Vaughn S Blackman BR . Distinct endothelial phenotypes evoked by arterial waveforms derived from atherosclerosis-susceptible and -resistant regions of human vasculature. Proc Natl Acad Sci U.S.A. (2004) 101:14871–6. doi: 10.1073/pnas.0406073101 PMC522013 15466704

